# Hospitalisation Utilisation and Costs in Schizophrenia Patients in Finland before and after Initiation of Risperidone Long-Acting Injection

**DOI:** 10.1155/2012/791468

**Published:** 2012-05-07

**Authors:** Christian Asseburg, Michael Willis, Mickael Löthgren, Niko Seppälä, Mika Hakala, Ulf Persson

**Affiliations:** ^1^ESiOR Oy, Tulliportinkatu 2, 70100 Kuopio, Finland; ^2^The Swedish Institute for Health Economics (IHE), 22002 Lund, Sweden; ^3^Amgen (Europe) GmbH Dammstrasse 23, P.O. Box 1557, CH-6301 Zug, Switzerland; ^4^Department of Psychiatry, Satakunta Hospital District, 29200 Harjavalta, Finland

## Abstract

*Objectives*. Quantify changes in hospital resource use in Finland following initiation of risperidone long-acting injection (RLAI). *Materials and Methods*. A retrospective multi-center chart review (naturalistic setting) was used to compare annual hospital bed-days and hospital episodes for 177 schizophrenia patients (mean age 47.1 years, 52% female, 72% hospitalized) before and after initiation of RLAI (between January 2004 and June 2005) using the within-patient “mirror-image” study design. The base case analytical approach allocated hospital episodes overlapping the start date entirely to the preinitiation period. In order to investigate the impact of inpatient care ongoing at baseline, the change in bed-days was also estimated using an alternative analytical approached related to economic modelling. *Results*. In the conventional analysis, the mean annual hospitalisation costs declined by €11,900 and the number of bed-days was reduced by 40%, corresponding to 0.19 fewer hospital episodes per year. The reductions in bed-days per patient-year were similar for patients switched to RLAI as inpatients and as outpatients. In the modelling-based analysis, an 8% reduction in bed-days per year was observed. *Conclusion*. Despite uncertainty in the choice of analytic approach for allocating inpatient episodes that overlapping this initiation, consistent reductions in resource use are associated with the initiation of RLAI in Finland.

## 1. Introduction

Schizophrenia is a serious mental illness causing significant social or occupational dysfunction. With an annual global incidence of 8 to 40 individuals per 100,000 per year [1], the total costs of treating schizophrenia are high [2] and may be as much as 3% of all health expenditures [3]. Most of the direct costs of schizophrenia (79%) result from hospitalisation or other residential care [3], thus a principal aim of treatment in schizophrenia patients is to prevent relapse, reduce the requirements for in-hospital treatment, and enable patients to lead near-normal lives.

Pharmacological treatments for schizophrenia have been available since the mid-1950s. The first class of medication, “typical antipsychotics,” is effective at treating psychotic symptoms but, while still used widely, is associated with problematic extrapyramidal side effects. The second generation of drugs, “atypical antipsychotics,” became available in the 1990s and may cause fewer extrapyramidal side effects, though medication noncompliance continues to be common in schizophrenia patients in part because of serious metabolic concerns. Long-acting depot formulations of some first-generation antipsychotic agents have been developed to improve medication compliance by shifting responsibility from unpredictable patients to their health care providers [2], and the extent to which long-acting injectable medication can reduce rates of relapse and rehospitalisation is a topic of active research [4].

Risperidone long-acting injection (RLAI, available in many countries as RISPERDAL CONSTA) is the first long-acting depot formulation of an atypical antipsychotic and was introduced in Finland in February 2003. Administered once every two weeks by intramuscular injection, RLAI provides steadier plasma levels than oral formulations and hence a more consistent relief of symptoms and lower rates of side effects [3].

While the unit cost of RLAI is higher than the unit costs of alternative antipsychotics at comparable doses, RLAI may provide a number of potentially offsetting economic benefits (in particular, reduced healthcare costs related to relapse and hospitalisation [3]). Indeed, a number of studies have identified reductions in hospital admission rates following initiation of risperidone in a host of countries. For example, Eriksson and colleagues [5, 6] found a 65% reduction in annual inpatient bed-days in Sweden; Chue and colleagues [7] found that the number of patients requiring hospitalization decreased continuously from 38% in the 3 months prior to RLAI to 12% after 1 year of treatment in an international study; Fuller and colleagues [8] found reductions of 37% in psychiatric-related hospitalizations and 56% in psychiatric-related hospital bed-days in the USA; Niaz and Haddad [9] found roughly 50% reductions in both number of admission and hospital days per patient-year in the UK though Taylor et al. [10] found little difference; Su and colleagues [11] found reductions in hospital admissions of 55% and inpatient bed-days of 48% in Taiwan; Willis and colleagues [12] found reductions of 27% in hospitalization rate and 45% in hospital bed-day; Carswell and colleagues [13] found that patients in New Zealand had fewer hospital admissions but longer lengths of stay. Health care is setting specific, however, and the economic efficiency of interventions in one country is seldom generalizable to another. We are not aware of data for Finland.

## 2. Aims of the Study

This study aim is to estimate the change in resource usage, hospitalisation rates, and costs seen in actual practice in Finland for schizophrenia patients before and after initiation of treatment with RLAI.

## 3. Patients and Methods

### 3.1. Study Design

We collected data using a retrospective, observational review of patient charts for patients suffering from schizophrenia or schizoaffective disorders and treated with RLAI in Finland. To estimate the changes in resource use associated with RLAI treatment, data for the periods before and after initiation are compared on a patient-by-patient within-subject basis (sometimes labelled “mirror-image” analysis [14]).

### 3.2. Endpoints

Outcome measures include the differences in the mean number of inpatient hospital bed-days per year, the mean rate of hospitalisation per year, and the mean annual costs of inpatient hospital care related to schizophrenia (reported in 2007 €).

### 3.3. Ethics Approval

Ethics approval was sought from the ethics review board at the Joint Municipal Authority for Medical and Social Services in North Karelia and granted. Informed consent was not required.

### 3.4. Patient Selection

Based on participation in previous studies or because they were known to use RLAI, 10 Finnish psychiatry sites were selected to participate: Pohjois-Karjala central hospital (Paihola), Harjavalta psychiatric hospital (Harjavalta), Pori mental health care centre (Pori), Turku city psychiatry department (Turku), Kaivanto psychiatric hospital (Kangasala), TaUCH Pitkäniemi hospital (Pitkäniemi), Helsinki and Uusimaa hospital district area Kellokoski psychiatric hospital (Kellokoski) and Tammiharju psychiatric hospital (Tammisaari), Kitee mental health care centre (Kitee), and Helsinki city psychiatric department (Helsinki).

Patients who participated in any RLAI clinical trial were excluded from this study. The inclusion criteria were at least 18 years old, diagnosed with schizophrenia or schizo-affective disorder, first initiation of RLAI treatment between 1 January 2004 and 30 June 2005, and medical chart record covering at least two years prior to RLAI treatment and at least until 31 July 2006. At each study site, investigators aimed to enlist all eligible subjects up to a maximum of 40 (to prevent overrepresentation of some sites).

### 3.5. Data Analytic Procedures

Single hospital episodes were recorded in the data collection forms using dates of admission and discharge. Hospitalisations that involved changes in level of care (psychiatric, psychiatric intensive, and forensic) were recorded in the hospital charts as a sequence of separate “episodes” with individual dates of admission and discharge. We merged these into a single episode except when calculating hospital costs.

When attributing observed resource use to RLAI or the prior treatment, hospitalisation episodes that overlapped the index initiation RLAI must be allocated to the periods before and after the index initiation. Given the seriousness of schizophrenia, withdrawal effects associated with the previous drugs [15], and time to adequate blood serum levels for most depot formulations (RLAI reaches satisfactory blood serum levels first after 3 weeks, until which time additional antipsychotic treatment is required to maintain adequate control [16]), an improvement sufficient for discharge to occur instantaneously after RLAI initiation cannot be expected. An intent-to-treat definition based strictly on the day of initiation, thus, would artificially penalise the RLAI by attributing the consequences of the failure of previous medications (and consequent poor health requiring hospitalization) to the postinitiation treatment.

**Figure 1:**
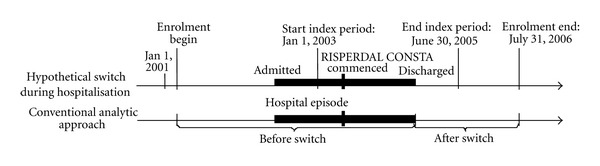
Illustration of the conventional allocation rule for hospitalisation episodes ongoing at the time of initiation. The start of the post-initiation period is adjusted so the entirety of hospitalisations episode overlapping the initiation date are included in the pre-switch period.

We adopt the simple allocation rule used by Fuller and colleagues [8], Niaz and Haddad [9], and others, who assigned the entire episode of hospitalisations ongoing at the time of initiation to the treatment ongoing at admission (“conventional analytic approach,” Figure 1). This approach seems reasonable because hospital resource use is more likely attributable to the treatment that was ongoing and failing at admission (possibly due to insufficient efficacy, sideeffects, or low adherence) than to a treatment that was begun after admission to hospital. The start of the exposure period to RLAI in a patient hospitalised when initiating RLAI is then adjusted to the date of hospitalisation episode discharge. Conservatively, patients who were initiated on RLAI as outpatients are assumed to start the post-initiation period at the actual time of initiation. The end of the exposure period is at death or study completion (31 July 2006), whether or not the patient was still taking RLAI. The control period runs from 2 years prior to treatment initiation with RLAI to the start of the treatment exposure period. 

By allocating to the prior treatment the entirety of the hospitalisation episodes that overlap the initiation, the conventional analytic approach may bias the results in favour of RLAI. This bias is stronger in proportion to the prevalence and length of overlapping hospitalisation episodes and ameliorated by longer study follow-up durations. Because this allocation affects only measures of resource use on-going at the time of initiation (bed-days and hospital costs), whereas a hospitalisation event is clearly before or after initiation, this source of bias does not apply to the endpoint of hospitalisation rates.

To investigate the sensitivity of study results to this allocation rule, we conducted two additional analyses. First, the primary endpoints are presented separately for patients who were hospitalised and were not hospitalised at the time of initiation. Results for the subgroup who started RLAI as outpatients are unaffected by this bias. Second, we present an alternative approach in which we model the change based on the fact that the number of bed-days per year is the product of annual hospitalisation rate and average length of inpatient stay per episode. Specifically, we multiply the empirical estimates of hospitalisation rates before and after the initiation of RLAI by the average lengths of stay per hospitalisation episode *strictly before* and *strictly after* initiation. This modelling-based estimate of resource use eliminates the effect of hospitalisation episodes ongoing at the time of initiation.

In analyses of inpatient bed-days, number of episodes, and hospitalisation costs, the unit of analysis is the patient. Results are presented as mean, standard error, and two-sided 95% confidence intervals. A difference was interpreted as statistically significant if the confidence interval for the estimated mean difference did not contain 0. To estimate the costs of hospital resource use, the recorded durations of episodes were multiplied by unit costs for bed-days in the different levels of inpatient service [17, 18]. Adjusted to 2007 € prices using Finnish price indices, the costs per bed-day are €280.46 for standard psychiatric care, €542.48 for psychiatric intensive care, and €510.00 for forensic care.

Additional subgroup analyses were carried out with regard to type of previous antipsychotic treatment based on recorded treatments on the day of initiation of RLAI or the previous day. Patients are classified into the following five mutually exclusive categories: typical oral only, typical depot only, atypical oral only, combination of any of these, or untreated (which does not necessarily imply drug-naïve).

## 4. Results

### 4.1. Descriptive Results

Data were collected for 199 patients. Twenty-two subjects were excluded from analysis because they were hospitalised before the start of RLAI and not discharged before the end of follow-up (2 patients), the start date of RLAI could not be established (*n* = 6), their RLAI prescription record was incomplete or inconsistent (*n* = 5), they started RLAI before 1 January 2004 (*n* = 2), or the number and duration of hospitalisation episodes could not be clearly established (*n* = 9). Multiple exclusion criteria applied to 2 patients. The analysis sample, thus, consists of 177 patients. 

Descriptive statistics for the patient population at the time of initiation to RLAI are presented in Table 1. Mean age at initiation of RLAI therapy was 47.1 years, 52% of the sample was female, and mean disease duration was 15.3 years. Patient functioning was poorly documented in hospital charts. Indeed, only 21 had a recorded Global Assessment of Functioning (GAF) score, only 49 had a recorded Global Assessment Scale (GAS) score, and only 27 had a recorded Clinical Global Impressions Scale (CGIS) score, and 92 patients had neither.

The lengths of the coverage periods and hospitalisations are presented in Table 2. By definition, two years of data on each patient are available prior to the initiation. Postinitiation data coverage ranges from 0.43 years (five patients died during study follow-up) to 2.56 years, with a mean of 1.80 years. 576 hospitalisation episodes were recorded during the study, of which 227 were entirely before the initiation of RLAI and 221 were entirely after the initiation. 128 patients (72%) were hospitalised when initiated on RLAI therapy. The mean duration of hospitalisations overlapping the index initiation was 120 days, considerably longer than the mean duration of hospital episodes observed strictly before (46 days) or strictly after initiation (53 days).

Statistics on the use of RLAI are detailed in Table 3. The most frequently cited reasons for initiating therapy with RLAI were noncompliance on other medications (63%), lack of efficacy on other medications (34%), and relapse (27%). The average duration of RLAI treatment observed during the study period was 1.33 years, ranging from 0 days (i.e., first and last dose occurring on the same day) to 2.56 years. For 66 patients, RLAI therapy was discontinued during the study follow-up, primarily for the following reasons: lack of efficacy (35%), non-compliance (35%), and patient choice (33%).  Table 4 shows the distribution of RLAI dosing over time. Seventy-six percent of patients started at 25 mg, doses above 50 mg were rarely observed.

### 4.2. Main Analysis

The results of the conventional analytic approach are shown in Table 5. The mean number of bed-days per patient per year was reduced by 24.89 bed-days (40%), from 62.89 to 38.00 per patient-year, a statistically significant difference. The mean number of hospitalisations per year was reduced by 0.19 episodes (20%), a statistically significant reduction from 0.93 episodes per year before initiation of RLAI to 0.74 episodes after. Statistically significant reductions in total hospitalisation costs are also associated with the initiation of RLAI therapy. Mean hospital costs per patient-year decreased by €11,948 (43%), from €28,046 per year before initiation of RLAI to €16,098 after initiation.

Table 6 presents results for the subgroups of patients who were initiated on RLAI during a hospitalization (*n* = 128) or on an outpatient basis (*n* = 49). Statistically significant reductions of 21.81 (36%) and 32.93 (48%) in the mean number of bed-days per year were observed in the inpatient and outpatient cohorts, respectively. A statistically significant reduction of 0.24 episodes (24%) in the mean number of hospitalisations per patient-year was observed in the inpatient subgroup; a reduction of 0.06 episodes (8%) in the outpatient subgroup did not reach statistical significance due to small sample size. For the modelling-based approach, the estimates of episodes per year in the two periods (0.93 and 0.74 episodes per patient-year, resp.) are multiplied with the average lengths of stay associated with hospitalisation in the two periods, excluding any hospitalisation episodes on-going at the time of initiation (45.6 and 53.0 days, resp.). This complementary approach estimates a reduction in the mean number of bed-days per patient per year by 3.2 (i.e., 8%), from 42.4 to 39.2 bed-days per patient-year.

Results by previous treatment are presented in Table 7. The results are suppressed for patients treated exclusively with typical antipsychotic agents just prior to the initiation as sample sizes were small. The combination therapy subgroup is made up predominantly of patients who receive atypical and typical oral agents in combination.

In the large subgroups of patients with previous atypical oral agent (alone or in combination), results are generally similar to the overall results, for example a reduction of 0.22 episodes in the single atypical oral subgroup, from 0.90 episodes per year before initiation to 0.69 episodes per year after the initiation of RLAI. For the small subgroup of patients untreated at the time of initiation (*n* = 9), the annual number of hospitalisation episodes before the initiation is estimated to be higher (1.18 per year) than the overall average, and a larger reduction by 0.77 is seen with the initiation to a postinitiation mean annual number of 0.41 hospitalisations. Resource use as measured by bed-days per year is, however, below average for this subgroup, with mean 50 days per year prior to the initiation and 18 days after initiation.

## 5. Discussion

Results suggest that RLAI is associated with sizeable and statistically significant reductions in resource use. The mean number of hospitalisations per year decreased by 20%. This translated into reduced inpatient bed-days and corresponding costs, but the magnitude is sensitive to the method of allocating hospitalisation episodes on-going at the time of initiation. The conventional approach resulted in a reduction in bed-days per patient-year by 40% and a corresponding cost-saving of over €16,000 per patient-year, while the alternative modelling-based approach found a reduction in inpatient days of 8%. The subgroup of patients who were initiated on RLAI on an outpatient basis (and which does not suffer from uncertainty about the appropriate allocation of hospital episodes overlapping the initiation) experienced a 48% reduction of bed-days per patient-year (similar to the conventional approach). Despite variation in the magnitude of this reduction between the three approaches, all analyses point to a consistent and considerable reduction in resource use associated with the initiation to RLAI. 

### 5.1. Study Strengths and Weaknesses

This study has some important strengths. First, the use of a noninterventional design allowed us to answer questions about “real-world” resource use in a timely manner. Through the use of chart review, resource use in schizophrenia patients could be observed as it occurred in actual practice rather than in the context of controlled clinical trials with corresponding protocol bias [11]. Moreover, the study endpoints are highly likely to be captured in patient charts, resulting in data integrity on hospitalisation episodes and costs.

Second, the retrospective study design was chosen to ensure recruitment of a reasonably-sized patient sample within the time constraints imposed by demands of the Finnish Pharmaceuticals Pricing Board (Lääkkeiden Hintalautakunta), which would have been impossible to achieve in a prospective study. With a relatively long patient follow-up of, on average, 1.8 years after initiation of RLAI, this study may also be more likely to capture the effects of treatment persistence and compliance, which a shorter study is likely to underestimate [4]. The “mirror-image” design, moreover, provides a within-group comparison that controls for time-invariant individual patient covariates.

Third, informed consent was not required. This may have avoided the selection bias related to willingness to participate in clinical studies [19] and facilitated the recruitment of a relatively large sample. Moreover, because the data before the initiation of RLAI are compared with data from the same patients after initiation, the study design controls for biases due to time-invariant individual covariates, irrespective of whether these are recorded in the study.

There are also several important study limitations. First, “mirror-image” studies are subject to inherent biases [14]. Because the treatment under investigation (RLAI) is typically initiated during an acute episode when patient health was sufficiently poor to necessitate a treatment initiation, gravitation to the mean [20] may have contributed to optimistic estimates in this study. The asymmetrical definition of treatment periods may have introduced a conservative bias because the dose titration period is included in the estimation of the RLAI treatment effect. The strict temporal sequence of periods before and after the initiation of RLAI may have caused biases relating to disease progression or changes in the health-care system (period bias), such as the conservative bias associated with the worsening severity status in schizophrenia patients over time. While the lack of a concurrent control group precludes assessment of these biases, leading some authors to consider results from “mirror-image” studies inconclusive [21], this design is nonetheless often used in studies of health resource use [14].

Second, this study may not represent schizophrenia patients generally because it appears to have recruited, on average, quite severely ill schizophrenia patients. Indeed, a “first-wave” effect, in which severely ill patients are more likely than others to receive a novel drug, may have occurred because the recruitment index period began soon after the launch of RLAI in Finland. Seventy-two percent were initiated on RLAI while hospitalised, which indicates above-average severity in the sampled schizophrenia patients. Treatment success may be less likely in these difficult-to-treat patients, so the resource use reductions reported here for the subgroup of patients initiating RLAI as outpatients may be more representative of the broader population of schizophrenia patients than the overall results reported here.

Third, controversy exists regarding the appropriate definition of treatment periods. Gianfrancesco et al. [14] argue that a pure intent-to-treat principle cannot and should not be applied to “mirror-image” studies because the before-treatment period is not defined according to intent-to-treat. This is a clear deficit inherent in the “mirror-image” study design. Health-economic studies, however, aim to compare the patient outcomes resulting from the decision to start different therapies, rather than during a treatment-specific period in which treatment is considered “successful.” It is therefore appropriate to follow the pragmatic, intent-to-treat principle when analysing health-economic datasets in general [22] and in schizophrenia [23]. The conventional analytic approach adjusts the initiation date in some patients and its results are thus, strictly speaking, not associated with the start of RLAI therapy. The subgroup results presented for patients initiating RLAI as outpatients are consistent with the intent-to-treat principle in the postinitiation period.

Fourth, there is no comparison with relevant treatment alternatives. Choosing a relevant comparator is difficult because typical depots differ from atypical depots in their sideeffects profile, atypical oral preparations differ in patient compliance with the treatment [24], and RLAI is the first-to-market atypical depot.

Fifth, there is methodological uncertainty about how to analyse hospitalisation episodes that are ongoing at initiation. Specifically, the entirety of these hospitalizations that overlapped the index initiation of RLAI could be allocated to the preinitiation treatment as in the conventional analytic approach in this study as well as elsewhere [5, 6, 8]. Other studies have excluded patients who initiated on an inpatient basis [10, 12] or carried out sensitivity analyses in which they were excluded ([5, 8] and here). The magnitude of treatment effect appears sensitive to analysis methods, which is significant here because nearly 75% of the sample falls into this category and because these hospital stays were longer (mean 120 days) than other stays on average (around 50 days).

Many of the above design weaknesses are due to a lack of consensus on the correct method for analysing resource use episodes that overlap the initiation date. Ideally, these limitations would be overcome in future studies by comparing data on the initiation of RLAI with data on the initiation of another therapy, suitably chosen to avoid introducing patient selection biases. The biases introduced by choice of method would then affect both arms equally so that the conclusions should be more robust to the chosen methods of analysis and allocation. Additionally, studies with longer follow-up durations would reduce the impact of the methodological uncertainty associated with attribution of the episode ongoing at initiation because its contribution to overall resource use will be smaller. Health-economic analysis would benefit from both innovations because long-term comparative results on the different consequences of relevant treatment options are of more use to decision makers than studies of single-treatment options.

### 5.2. Interpretation of Study Results

Several other studies have collected data on resource use in patients diagnosed with schizophrenia or schizoaffective disorder and treated with RLAI. The results of the conventional analytic approach in the present study appear generally consistent with other studies (accounting for some differences in the design and recruitment) [6, 8, 10–13]. The similarities with an identically designed study in neighbouring Sweden is striking, 40% fewer bed-days in the current study and 45% in Willis et al. [12]. 

While the present study was not designed to investigate the reasons for reduced resource use in patients being treated with RLAI, the large proportion of patients who were initiated on RLAI because of noncompliance with the previous treatment (63%) suggests that doctors choose RLAI to improve medication compliance, a likely benefit of RLAI compared to oral medication [3].

## 6. Conclusion

This study found consist evidence that sizable reductions in resource usage are associated with the initiation of RLAI in Finland. The choice of analytic approach for allocating inpatient episodes that are ongoing at the initiation of therapy affects estimates of the magnitude, however, and future work to evaluate a novel approach based on economic modelling is desired.

## Figures and Tables

**Table 1:** Patient characteristics at time of initiation of RLAI.

Variable	*N*	Mean
Age (years)	177	47.1 (SD 13.6) Range 22–84
Female (%)	177	92 (52.0%)
Disease duration		
Years	137*	15.3 (SD 12.5)
% less than 4 years	137*	31 (17.5%)
% 4 to 10 years	137*	44 (24.8%)
% more than 10 years	137*	102 (57.7%)
GAF (raw score)	21*	22.5 (SD 13.9) Range 5–60
GAS (raw score)	49*	35.2 (SD 9.6) Range 19–58
CGI-S (%)		
Normal	177	0
Borderline or mildly Ill	177	1 (0.6%)
Moderately Ill	177	2 (1.1%)
Markedly Ill	177	16 (9.0%)
Severely or among most extensively Ill	177	8 (4.5%)
Unknown	177	150 (84.7%)
Occupational status (%)		
Full time	177	2 (1.1%)
Part time	177	0
Sheltered work	177	1 (0.6%)
Unemployed	177	13 (7.3%)
Retired	177	121 (68.4%)
Long-term sick leave	177	33 (18.7%)
Other	177	7 (4.0%)
Unknown	177	0
Accommodation status (%)		
Psychiatric nursing home	177	15 (8.5%)
Sheltered living	177	5 (2.8%)
With parents or relatives	177	20 (11.3%)
Own apartment	177	125 (70.6%)
Other	177	12 (6.8%)
Unknown	177	0

*Missing information resulted in reduced sample sizes.

**Table 2:** Length of follow-up and of hospitalisation episodes.

Variable	*N*	Mean	Standard deviation	Min	Max
Follow-up prior to initiation of RLAI (years)	177	2.00	—	—	—
Follow-up following initiation of RLAI (years)	177	1.80	0.45	0.43	2.56
Proportion of patients hospitalised at initiation of RLAI	177	127 (72%)	—	—	—
Hospital length of stay (days per episode)					
All Episodes	576	65.0	95.4	0*	816
Episodes strictly before RLAI initiation	227	45.6	53.2	1	405
Episodes overlapping RLAI initiation	128	120.1	145.0	9	816
Episodes strictly after RLAI initiation	221	53.0	80.3	0*	638

*Inpatient hospitalisation episodes that recorded discharge on the day of admissions were assigned a duration of zero days.

**Table 3:** Use of RLAI.

Variable	*N*	Mean
Reasons for initiation to RLAI* (%)		
Adverse event: weight gain	177	3 (1.7%)
Adverse event: extrapyramidal symptoms	177	14 (7.9%)
Adverse event: other	177	11 (6.2%)
Lack of efficacy	177	61 (34.5%)
Relapse	177	47 (26.6%)
Patient choice	177	26 (14.7%)
Noncompliance	177	112 (63.3%)
Unknown	177	0
Duration of RLAI use (days)	177	485.5 (SD 287.4) range 0–936
RLAI persistence (proportion of patients known to continue therapy during)		
at least 6 months	175^†^	134 (76.6%)
at least 12 months	172^†^	122 (70.9%)
at least 18 months	128^†^	87 (68.0%)
at least 24 months	68^†^	45 (66.2%)
Reasons for stopping RLAI* (%)		
Adverse event: weight gain	66	2 (3.0%)
Adverse event: extrapyramidal	66	3 (4.5%)
Adverse event: other	66	10 (15.2%)
Lack of efficacy	66	23 (34.8%)
Relapse	66	2 (3.0%)
Patient choice	66	22 (33.3%)
Noncompliance	66	23 (34.8%)
Unknown	66	0

*Multiple answers were allowed. ^†^Reduced sample size reflects follow-up durations.

**Table 4:** RLAI dose distributions in patients beginning with RLAI, and every 6 months onwards. for each patient, the last known observation on dose was carried forward to the time point. Two patients were excluded from the analysis of dose changes at later time points because of missing information.

	At treatment beginning	6 months later	12 months later	18 months later	24 months later
*N* (patients)	177 (100%)	132 (100%)	121 (100%)	87 (100%)	45 (100%)
25 mg	134 (76%)	41 (31%)	33 (27%)	24 (28%)	17 (38%)
37.5 mg	24 (14%)	40 (30%)	35 (29%)	26 (30%)	13 (29%)
50 mg	19 (11%)	48 (36%)	49 (40%)	34 (39%)	14 (31%)
62.5 mg	0	1 (1%)	1 (1%)	0	0
75 mg	0	2 (2%)	3 (2%)	3 (3%)	1 (2%)
100 mg	0	0	0	0	0

**Table 5:** Results of the main analysis. Hospitalisation episodes overlapping the date of RLAI initiation are allocated to the period before initiation. Sample unit (*N*) is the patient.

Endpoints	Before initiation		After initiation		Difference	
	Mean (SE)	95% CI	Mean (SE)	95% CI	Mean (SE)	95% CI
Inpatient bed-days per patient-year (*N* = 177)	62.89 (4.16)	(54.74; 71.04)	38 (5.19)	(27.83; 48.17)	−24.89 (5.93)	(−36.51; 13.26)
No. hospitalizations per patient-year (*N* = 177)	0.93 (0.05)	(0.83; 1.03)	0.74 (0.09)	(0.57; 0.91)	−0.19 (0.09)	(−0.36; −0.01)
Hospital costs per patient-year, € (*N* = 177)	28,046 (1,782)	(24,554; 31,539)	16,098 (2,117)	(11,949; 20,248)	−11,948 (2,555)	(−16,995; −6,941)
Cost of other antipsychotic agents, € per patient-year (*N* = 177)	907 (148)	(617; 1,197)	1,130 (174)	(788; 1,471)	233 (135)	(−43; 488)
Cost of RLAI per patient-year, € (*N* = 177)	—	—	4,193 (196)	(3,809; 4,576)	—	—

**Table 6:** Results of the subgroup analysis for the endpoints: inpatient bed-days per patient and year, and number of hospitalisations per patient and year. Results are presented for subgroups of patients who are in- or outpatients at the time of initiating RLAI.

Endpoints	Before initiation		After initiation		Difference	
	Mean (SE)	95% CI	Mean (SE)	95% CI	Mean (SE)	95% CI
Inpatient bed-days per patient-year						
Inpatient (*N* = 128)	60.88 (4.03)	(52.97; 68.79)	39.07 (6.13)	(27.06; 51.09)	−21.81 (7.21)	(−35.94; −7.68)
Outpatient (*N* = 49)	68.13 (10.76)	(47.05; 89.22)	35.2 (9.82)	(15.96; 54.44)	−32.93 (10.23)	(−52.98; −12.98)
No. hospitalizations per patient-year						
Inpatient (*N* = 128)	0.99 (0.06)	(0.87; 1.11)	0.76 (0.11)	(0.54; 0.97)	−0.24 (0.11)	(−0.45; −0.02)
Outpatient (*N* = 49)	0.77 (0.09)	(0.59; 0.95)	0.71 (0.14)	(0.43; 0.98)	−0.06 (0.14)	(−0.34; 0.21)

**Table 7:** Results of the subgroup analysis for the endpoints: inpatient bed-days per patient and year, number of hospitalisations per patient and year, and hospital costs per patient and year. Results are presented for previous therapy subgroups containing more than 5 patients.

Endpoints	Before initiation		After initiation		Difference	
	Mean (SE)	95% CI	Mean (SE)	95% CI	Mean (SE)	95% CI
Inpatient bed-days per patient-year						
Atypical oral only (*N* = 129)	61.63 (4.30)	(53.20; 70.05)	37.69 (6.03)	(25.87; 49.51)	−23.94 (7.21)	(−38.07; −9.81)
Atypical oral Combination (*N* = 32)	70.76 (12.80)	(45.67; 95.86)	38.95 (13.27)	(12.95; 64.95)	−31.81 (14.35)	(−59.93; −3.98)
Untreated (*N* = 9)	50.06 (15.94)	(18.82; 81.30)	17.8 (6.65)	(4.77; 30.84)	−32.26 (16.19)	(−63.99; −0.54)
No. hospitalizations per patient-year						
Atypical oral only (*N* = 129)	0.9 (0.06)	(0.80; 1.01)	0.69 (0.09)	(0.51; 0.86)	−0.22 (0.09)	(−0.40; −0.03)
Atypical oral Combination (*N* = 32)	0.96 (0.14)	(0.69; 1.24)	0.78 (0.23)	(0.32; 1.24)	−0.18 (0.17)	(−0.51; 0.15)
Untreated (*N* = 9)	1.18 (0.30)	(0.59; 1.78)	0.41 (0.14)	(0.13; 0.68)	−0.77 (0.29)	(−1.34; −0.21)
Hospital costs per patient-year, €						
Atypical oral only (*N* = 129)	27,560 (1,934)	(23,770; 31,351)	16,177 (2,546)	(11,187; 21,167)	−11,383 (3,007)	(−17,276; −5,491)
Atypical oral Combination (*N* = 32)	32,415 (5,439)	(21,755; 43,075)	15,666 (5,027)	(5,812; 25,519)	−16,749 (6,782)	(−30,041; −3,457)
Untreated (*N* = 9)	20,561 (6,633)	(7,561; 33,562)	8,435 (3,379)	(1,812; 15,059)	−12,126 (7,735)	(−27,28; 3,035)
